# Physical Frailty is Correlated with Worse Quality of Life in Older Adults with Hypertension

**DOI:** 10.1093/geroni/igab046.3005

**Published:** 2021-12-17

**Authors:** Pan Liu, Yaxin Zhang, Shijie Li, Ying Li, Yumeng Chen, Ou Zhao, Yun Li, Lina Ma

**Affiliations:** Xuanwu Hospital, Capital Medical University, National Research Center for Geriatric Medicine, Beijing, Beijing, China (People's Republic)

## Abstract

Background: Hypertension is one of the commonest chronic cardiovascular diseases in older adults. Frailty and hypertension often coexist in older people, but few studies have explored frailty in older hypertensive adults. We aimed to explore the correlation of frailty with quality of life in older hypertensive adults. Method: We enrolled 291 patients with hypertension aged ≥60 years. Ambulatory blood pressure monitor was performed. Physical frailty was assessed by Fried phenotype. Quality of life was assessed by SF-36. Results: Forty-eight (16.5%) patients were frail. Compared with non-frail older hypertensive patients, frail patients were older, had lower education levels, a higher rate of living alone, and a longer duration of hypertension. Moreover, they had lower diastolic blood pressure (DBP) and mean arterial pressure (MAP), and higher pulse pressure, more chronic diseases, a higher proportion of calcium channel blockers (CCBs) usage, and worse quality of life. Frailty scores were positively correlated with pulse pressure, and negatively correlated with DBP and MAP. The SF-36 score was negatively correlated with frailty scores and positively correlated with grip strength and walking speed. After adjusting for age, the SF-36 score was negatively correlated with frailty and positively correlated with walking speed. Frailty, when adjusted for age, duration of hypertension, DBP and comorbidity, had a significant effect on the SF-36 score. Conclusion: Frailty was associated with worse quality of life of older adults with hypertension. Frailty prevention and intervention may help improve the quality of life of older hypertensive adults. Keywords: frailty, older adults, hypertension, quality of life

